# Gene Spectrum and Clinical Traits of Nine Patients With Oocyte Maturation Arrest

**DOI:** 10.3389/fgene.2022.772143

**Published:** 2022-01-24

**Authors:** Mingzhu Huo, Yile Zhang, Senlin Shi, Hao Shi, Yidong Liu, Lingyun Zhang, Yanchi Wang, Wenbin Niu

**Affiliations:** ^1^ Center for Reproductive Medicine, The First Affiliated Hospital of Zhengzhou University, Zhengzhou, China; ^2^ Henan Key Laboratory of Reproduction and Genetics, The First Affiliated Hospital of Zhengzhou University, Zhengzhou, China; ^3^ Henan Provincial Obstetrical and Gynecological Diseases (Reproductive Medicine) Clinical Research Center, The First Affiliated Hospital of Zhengzhou University, Zhengzhou, China; ^4^ Henan Engineering Laboratory of Preimplantation Genetic Diagnosis and Screening, The First Affiliated Hospital of Zhengzhou University, Zhengzhou, China

**Keywords:** female infertility, oocyte maturation arrest, genetic variants, clinical phenotype, zona pellucida

## Abstract

**Background:** Oocyte maturation arrest is a disease that produces immature oocytes and cannot be mature after culturing *in vitro*, which leads to female primary infertility. We aimed to summarize nine representative patients in our center to retrospectively analyze the genetic variants and clinical characteristics of oocyte maturation arrest.

**Methods:** This study examined and analyzed nine families with oocyte maturation arrest. Whole-exome sequencing (WES) of the probands was performed to detect the pathogenic variants. Sanger sequencing verified the WES findings in patients and available parents. ExAC database was used to search the variant frequency. The variants were assessed by pathogenicity and conservational property prediction analysis and according to the American College of Medical Genetics and Genomics (ACMG). Phenotypes of oocytes were evaluated by a light microscopy, and the phenotype-genotype correlation was also evaluated.

**Results:** Nine pathogenic variants in five genes were detected in nine patients, of which three were novel variants, including *PATL2* [c.1374A > G (p. Ile458Met)] and [1289-1291del TCC (p. Leu430del)] and *ZP2* [c.1543C > T (p. Pro515Ser)]. Nine variants were predicted to be pathogenic, resulting in different types of oocyte maturation arrest and clinical phenotypes.

**Conclusion:** Three novel pathogenic variants were identified, enabling the expansion of the gene variant spectrum. The related pathogenic mutations of the *PATL2*, *TUBB8*, and *ZP1∼3* genes were highly suggestive of being causative of oocyte maturation arrest.

## Introduction

Oocyte maturation is an important prerequisite for successful fertilization and embryonic development. Oocyte maturation includes cytoplasmic maturation and nuclear maturation (Swain and Pool, 2008). Cytoplasmic maturation is characterized by cytoplasmic changes required for cell fertilization, activation, and embryonic development. Furthermore, the sign of nuclear maturation is the rupture of germinal vesicles and the exclusion of the first polar body (Krisher, 2004; Sirard et al., 2006). Errors in any link may lead to oocyte maturation block, fertilization failure, and early embryo arrest, resulting in primary infertility. In 1990, Rudak et al. described the first patient of oocyte maturation arrest (Rudak et al., 1990). Depending on the stage of oocyte meiosis, there are four types of maturation failure, including arrest at germinal vesicle (GV), metaphase I (MI), and metaphase II (MII) and a mixed arrest with oocytes at multiple meiotic stages (Beall et al., 2010).

At present, there are six different types of oocyte maturation arrest in genetics. Oocyte maturation arrest type 1 (OMIM195000) is caused by *ZP1* mutation, leading to the loss of zona pellucida (ZP) (Huang et al., 2014). Type 2 (OMIM616768) is caused by *TUBB8* mutation. *TUBB8* encodes the main tubulin isotype that assembles the spindle of human oocytes. Once interrupted, abnormal spindles can be seen and the cleavage of oocytes terminates at the MI stage (Zhao et al., 2020). Type 3 (OMIM182889) is oocyte degeneration caused by zona pellucida deletion and ‘empty follicle syndrome (EFS)’ (Chen et al., 2017c), which was associated with *ZP3* gene mutation. EFS means *in vitro* fertilization (IVF) treatment. Although the number and size of follicles are normal, oocytes cannot be obtained after repeated ovarian stimulation (Coulam et al., 1986). Type 4 (OMIM614661) is mainly characterized by the arrest of oocyte development at the GV or MI stage, caused by *PATL2* mutation (Chen et al., 2017a). Type 5 (OMIM614084) associated with the *WEE2* gene mainly stagnates oocytes at the MII phase (Sang et al., 2018). Type 6 (OMIM182888) related to *ZP2* mutation results in female primary infertility due to abnormal ZP of oocytes, which consequently results in poor binding with spermatozoa (Dai et al., 2019).

In this study, we recruited nine families. All females had a history of primary infertility and were diagnosed with oocyte maturation arrest. We found nine pathogenic variants in five genes, including the *PATL2*, *TUBB8*, and *ZP1∼3* genes. We identified three novel mutation sites through whole-exome sequencing (WES) and analyzed the genetic causes of oocyte maturation arrest. All of these findings expand the genotypic spectrum of the *PATL2* and *ZP2* genes, which will lay the foundation for future genetic counseling.

## Materials and Methods

### Ethics Approval and Case Recruitment

This study was approved by the institutional review board (IRB) of the Center for Reproductive Medicine, The First Affiliated Hospital of Zhengzhou University (Ethic no. 2019-KY-166). All participants have provided written informed consent. We studied nine primary infertility females who were diagnosed with oocyte maturation arrest. Their parents were also studied for inheritance mode identification when available.

### Whole-Exome Sequencing

Genomic DNA (gDNA) was extracted with QIAamp DNA Blood Mini Kit (QIAGEN, Germany, 51306) from peripheral blood following the manufacturer’s instructions. gDNA was quantified with the Quant-iT dsDNA HS Assay Kit (Invitrogen, Carlsbad, CA). MGIEasy exon capture V5 probe kit (BGI, China, 1000007746) was used for library construction and target region capturing. Library quality was measured by both Qubit 4 (Thermo Fisher Scientific, USA) and Bioanalyzer 2100 (Agilent, USA). After quality control, the libraries were pooled and sequenced to paired-end 100 bp on the MGISEQ-2000 system (MGI Technology Ltd. Co., China).

### Variant Interpretation

PROVEAN (http://provean.jcvi.org), PolyPhen-2 (http://genetics.bwh.harvard.edu/), and SIFT (http://sift.jcvi.org) were used to predict the pathogenicity of the mutation site, and the ExAC (http://exac.broadinstitute.org/) database was used to search for the corresponding variant frequency. The conserved property of the variants was analyzed using UniProt (https://www.uniprot.org/). The pathogenicity interpretation of the variants followed the American College of Medical Genetics and Genomics (ACMG) recommendations.

### Evaluation of Oocyte and Embryo Phenotype

Oocytes were obtained from the patients undergoing IVF or intracytoplasmic sperm injection (ICSI). The morphology of oocytes and embryonic development were observed by a light microscope. We studied the relationship between phenotype and genotype by clinical tests and the clinical records of the patients.

## Results

### Mutational Spectrum and Phenotypes in *PATL2*

**FIGURE 1:**
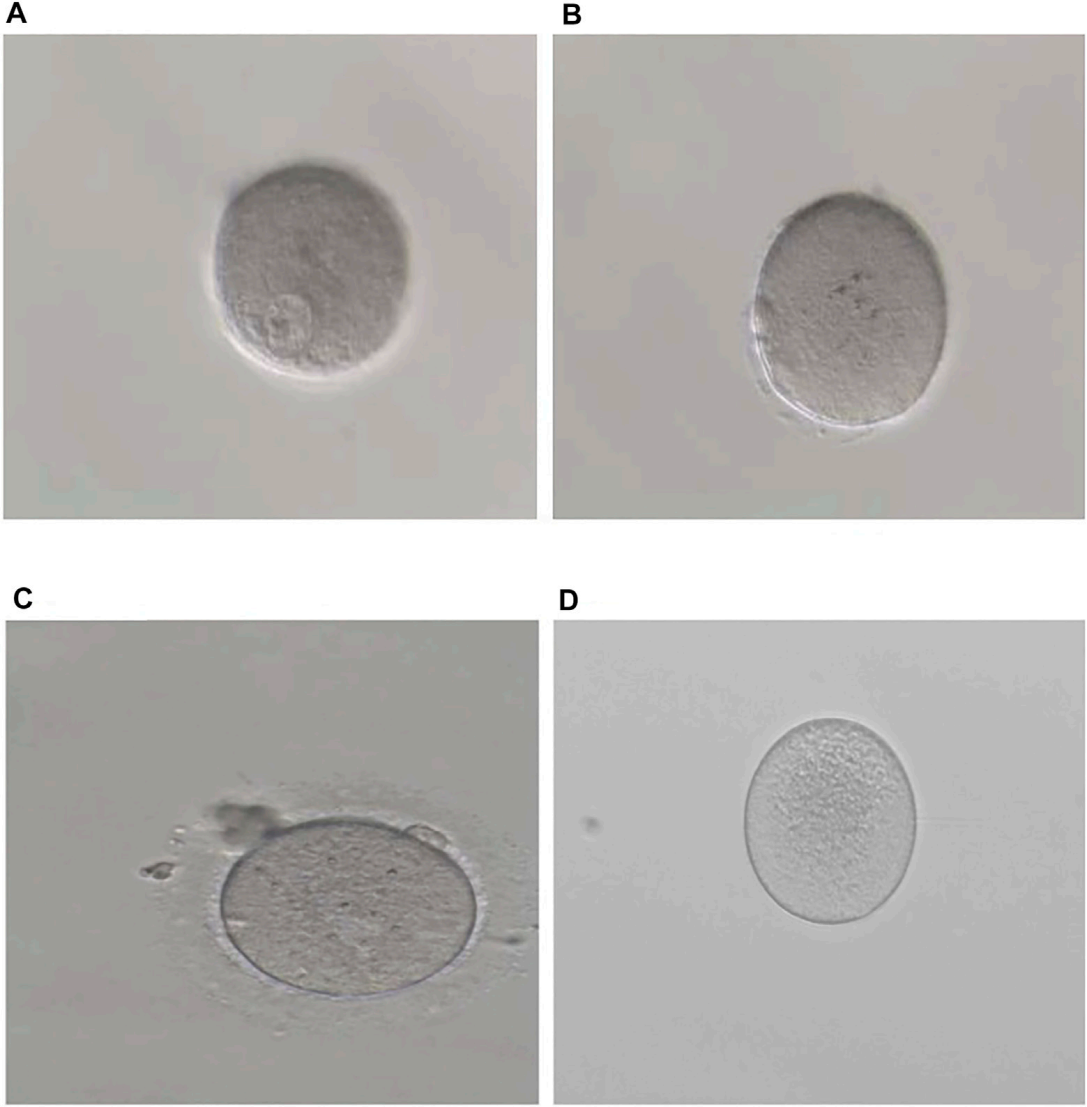
Phenotypes of oocytes from patients with maturation arrest. **(A)** Oocyte at GV (family 1). **(B)** Oocyte at MI (family 5). **(C)** Oocyte at MII (family 9). **(D)** Oocyte without ZP (family 7).

In family 1, the 32-year-old woman suffered from primary infertility for 10 years, although with a normal menstrual period and normal semen parameters for her partner. The patient experienced three IVF/ICSI cycles. Her first IVF treatment used an early-follicular phase long-acting gonadotropin-releasing hormone (GnRH) agonist long protocol, which captured eight oocytes: five were at the GV phase, two were at the MI phase, and one degenerated. Totally, 28 oocytes were obtained in the second ICSI treatment using a midluteal short-acting GnRH agonist long protocol, all of which were at the GV phase. In her third cycle, 16 oocytes were obtained using a modified early-follicular phase long-acting GnRH agonist long protocol, all of which were at the GV phase (Figure 1A). No embryos were available in the three IVF/ICSI cycles (Table 1).

**TABLE 1:** Clinical characteristics of patients and their retrieved oocytes.

Family	Age (years)	Duration of infertility (years)	Previous IVF/ICSI cycles	Total no. of oocytes retrieved	GV oocyte	MI oocyte	MII oocyte	Oocyte with abnormal morphology	Immature oocyte (unknown stage)	No. of usable embryos
1 (I458M)	32	10	3	52	49	2	0	0	0	0
(L430del)										
2 (V179M)	31	3	2	5	0	5	0	0	0	0
3 (V229A)	30	8	1	6	2	4	0	0	0	0
4 (V179M)	42	12	2	12	0	7	0	1	4	0
5 (S176W)	28	7	2	31	1	28	0	0	2	0
6 (Q292*)	27	6	2	7	4	1	0	2	0	1
7 (A134T)	33	3	2	3	0	1	0	2	0	0
8 (P515S)	32	4	2	20	14	5	0	1	0	0
9 (V255M)	31	2	1	10	0	9	1	0	0	0

IVF, *in vitro* fertilization; ICSI, intracytoplasmic sperm injection; GV, germinal vesicle; MI, metaphase I; MII, metaphase II.

**FIGURE 2:**
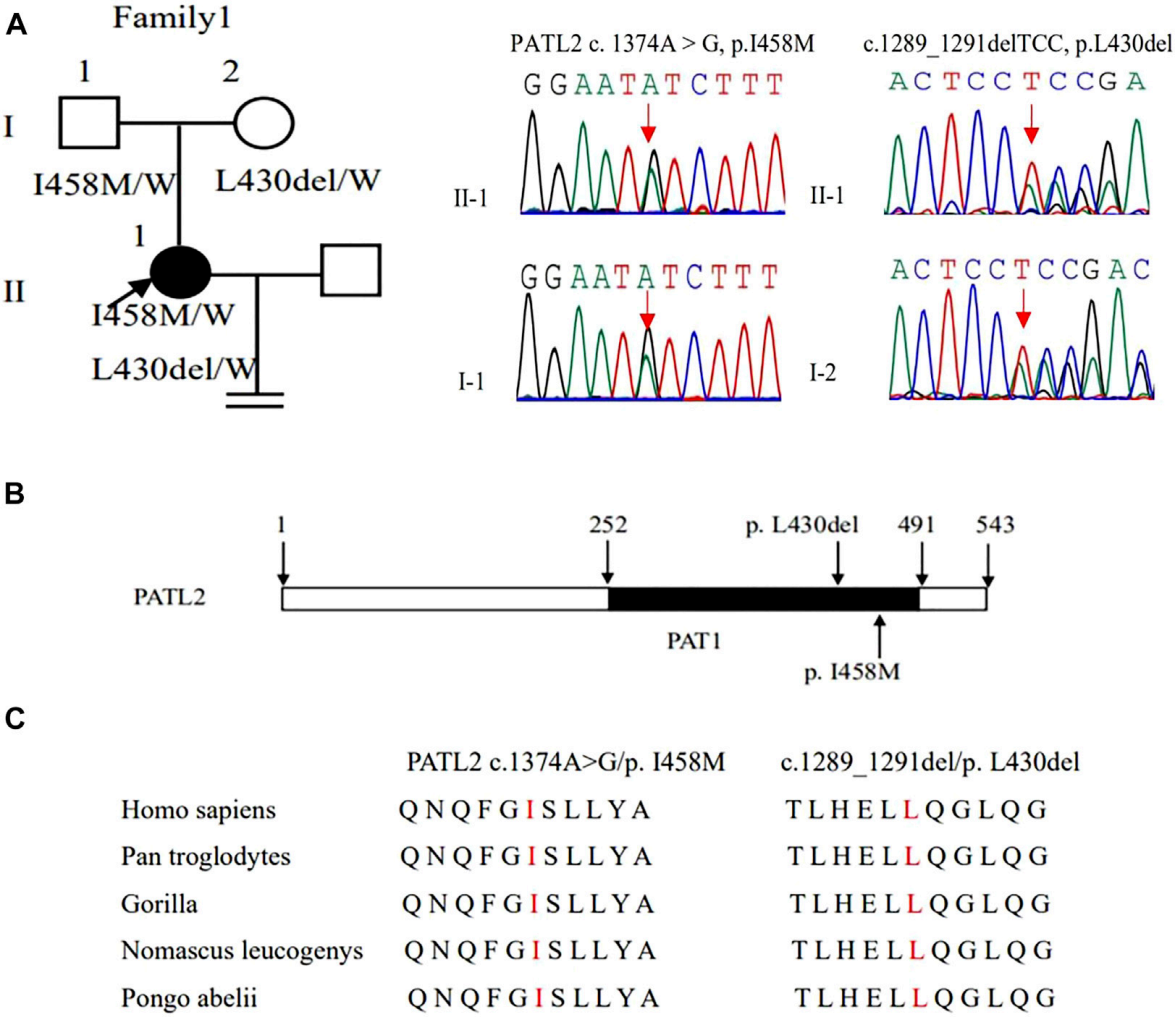
Genotypic features of family 1. **(A)** Pedigrees of family 1 with female infertility. Sanger sequencing confirmation is shown on the right of the pedigrees. The “=” sign indicates infertility, and black circles represent affected individuals. The “W” sign means wild type. **(B)** The positions of the novel mutations are indicated in the corresponding amino acids shown on the PATL2 protein. **(C)** Preservative mutation analysis for the novel sites in different species.

WES detected compound heterozygous variants c. 1374A > G (p. Ile458Met) and c.1289_1291delTCC (p. Leu430del) in the *PATL2* gene, and both variants were verified by Sanger sequencing. Additionally, we also identified that variants c. 1374A > G (p. Ile458Met) and c.1289_1291delTCC were inherited from the father and mother, respectively, by Sanger sequencing (Figure 2A). These two variants were neither reported in the HGMD database nor publications. According to ACMG guidelines, c. 1374A > G and c.1289_1291delTCC were classified as likely pathogenic (Table 2). *In silico* prediction by PolyPhen-2 and PROVEAN suggested that both variants are deleterious. The variants of *PATL2* were not reported in the ExAC browser (Table 3). The variant coordination and the conservation analysis among primate species are indicated in Figures 2B,C. The results showed that mutations in *PATL2* are highly evolutionarily conserved among different primate species.

**TABLE 2:** The evidence item description according to ACMG.

Family	Variant	Gene	Interpretation	Conclusion
1	c.1374A > G: p. I458M	*PATL2*	PM1^a^ + PM2^b^ + PP2^c^ + PP4^d^	Likely pathogenic
1	c.1289_1291delTCC: p. L430del	*PATL2*	PM1 + PM2 + PM4^e^ + PP4	Likely pathogenic
2	c.535G > A: p. V179M	*TUBB8*	PM1 + PM2 + PP2	Uncertain significance
3	c.686T > C: p. V229A	*TUBB8*	PM1 + PM2 + PP2 + PS3^f^ + PP1^g^ + PP4	Pathogenic
4	c.535G > A: p. V179M	*TUBB8*	PM1 + PM2 + PP2	Uncertain significance
5	c.527C > G: p. S176W	*TUBB8*	PM1 + PM2 + PP2 + PM5^h^	Likely pathogenic
6	c.874C > T: p. Q292*	*ZP1*	PVS1^i^ + PM2+PM3^j^ + PP4	Pathogenic
7	c.400G > A: p. A134T	*ZP3*	PS3+PS4_Supporting^k^ + PM2 +PP1_Moderate^l^	Pathogenic
8	c.1543C > T: p. P515S	*ZP2*	PM2	Uncertain significance
9	c.763G > A: p. V255M	*TUBB8*	PS4_Supporting + PM2+PM6^m^ + PP1_Moderate + PP2	Likely pathogenic

Note: the phenotype in the patient matches the gene’s disease association with reasonable specificity.

Note: pathogenicity classification has been made from a reputable source.

a The variation is located in a mutational hot spot.

b The frequency of the variant is less than 0.01 or absent from gnomAD, 1000 Genome Program, and ExAC databases.

c Missense variant in a gene that has a low rate of benign missense variation and where missense variants are a common mechanism of disease.

d Patient’s phenotype or family history is highly specific for a disease with a single genetic etiology.

e Protein length changes due to in-frame deletions in a non-repeat region or stop-loss variants.

f Well-established *in vitro* or *in vivo* functional studies supportive of a damaging effect on the gene.

g Co-segregation with a disease in multiple affected family members in a gene definitively known to cause the disease.

h Novel missense change at an amino acid residue where a different missense change determined to be pathogenic has been seen before.

i The nonsense variant in a gene where loss of function is a known disease mechanism.

j For recessive disorders, detected in trans with a pathogenic variant.

k The prevalence of the variant in affected individuals is significantly increased compared to the prevalence in controls.

l Co-segregation with disease in multiple affected family members in a gene definitively known to cause the disease. Note: it has stronger evidence with increasing segregation data.

m Assumed *de novo*, but without confirmation of paternity and maternity.

**TABLE 3:** Overview of related mutations in the 9 families.

Family	Gene	Genomic coordination	cDNA change	Protein change	Variant type	Inheritance	PROVEAN^a^	PolyPhen-2^b^	SIFT^c^	ExAC (total)^d^	ExAC (East Asian)^d^
1	*PATL2*	chr15:44959393	c.1374A > G	p. Ile458 Met	Missense	AR	N	PD	N	NA	NA
1	*PATL2*	chr15:44960613-44960616	c.1289_1291delTCC	p. Leu430del	In-frame deletion	AR	D	NA	NA	NA	NA
2	*TUBB8*	chr10:93797	c.535G > A	p. Val179Met	Missense	AD/AR	D	PD	D	8.322e-06	0
3	*TUBB8*	chr10:93646	c.686T > C	p. Val229 Ala	Missense	AD/AR	D	PD	D	NA	NA
4	*TUBB8*	chr10:93797	c.535G > A	p. Val179Met	Missense	AD/AR	D	PD	D	8.322e-06	0
5	*TUBB8*	chr10:93805	c.527C > G	p. Ser176Trp	Missense	AD/AR	D	PD	D	NA	NA
6	*ZP1*	chr11:60638477	c.874C > T	p. Gln292^e^	Nonsense	AR	D	NA	NA	8.238e-05	0.0001
7	*ZP3*	chr7:76058 919	c.400G > A	p. Ala134Thr	Missense	AD	D	PD	D	NA	NA
8	*ZP2*	chr16:21212841	c.1543C > T	p. Pro515Ser	Missense	AR/AD^e^	D	PD	NA	NA	NA
9	*TUBB8*	chr10:93569	c.763G > A	p. Val255Met	Missense	AD/AR	N	PD	D	6.77e-05	0

AD, autosome dominant; AR, autosome recessive; N, neutral; D, deleterious; NA, not available.

a Variant effect predicted by PROVEAN.

b Variant effect predicted by PolyPhen-2.

c Variant effect predicted by SIFT.

d Frequency of corresponding variants in the total and East Asian population of ExAC.

e OMIM database shows that *ZP2* follows a recessive inheritance pattern and a recent study found it was also inherited in an autosomal dominant pattern (Yang et al., 2021).

### Mutational Spectrum and Phenotypes in *TUBB8*

In family 2, the patient was a 31-year-old woman with a three-year history of primary infertility. In her two IVF cycles, five oocytes were retrieved, all at the MI stage, and failed to be fertilized. In family 4, the 42-year-old woman had a 12-year history of primary infertility. The patient underwent two IVF/ICSI cycles. In her first IVF cycle, an early-follicular phase long-acting GnRH agonist long protocol was adopted, eight oocytes were retrieved: seven were at MI and one was with abnormal morphology. In the second attempt with a midluteal short-acting GnRH agonist long protocol, four immature oocytes were obtained (unknown stage) as shown in Table 1.

**FIGURE 3:**
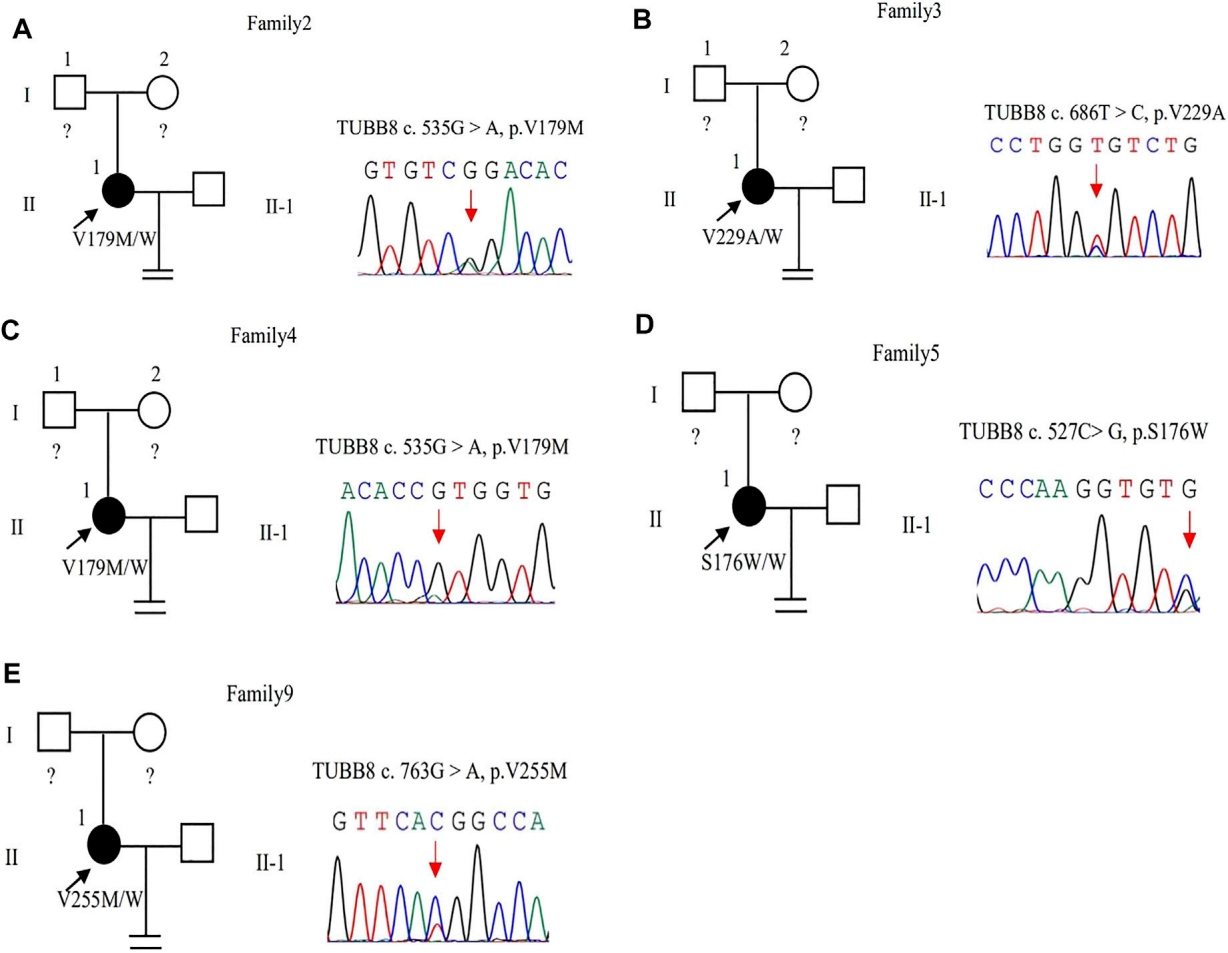
Pedigrees of 5 families with *TUBB8* variants. Sanger sequencing confirmation is shown on the right of the pedigrees. The “=” sign indicates infertility, and black circles represent affected individuals. The “W” sign means wild type, and question marks indicate the absence of a DNA sample. **(A–E)** represent different families.

The same variant c.535G > A (p.Val179Met) in exon 4 of the *TUBB8* gene was found in these two patients by WES (Figures 3A,C). The site of c.535G > A was classified as an uncertain significance variant according to the ACMG guidelines (Table 2). The results of PROVEAN, PolyPhen-2, and SIFT prediction suggested that the mutation was pathogenic (Table 3).

In family 3, a 30-year-old woman had primary infertility for eight years. She underwent one ICSI cycle, which used a progestin-primed ovarian stimulation. Six oocytes were retrieved, including two GV and four MI oocytes (Table 1). All oocytes had fertilization failure because they were immature even after culturing 24 h *in vitro*. The WES detected variant c.686T > C (p.Val229Ala) in exon 4 of the *TUBB8* gene (Figure 3B). The variant was interpreted as pathogenic according to the ACMG guidelines (Table 2). The results of PROVEAN, PolyPhen-2, and SIFT prediction suggested that the mutation was pathogenic (Table 3).

In family 5, the patient was a 28-year-old woman with a seven-year history of primary infertility. In the first IVF cycle, after an early-follicular phase long-acting GnRH agonist long protocol, 13 oocytes were retrieved, among which 11 were at the MI stage and 2 were immature oocytes (unknown stage). One oocyte developed into two pronuclei after fertilization, but no usable embryo was formed finally. In her second attempt, by a midluteal short-acting GnRH agonist long protocol, 18 oocytes were retrieved: one was at the GV stage and the rest were at the MI stage. They remained unmatured even after *in vitro* maturation (IVM) for 24 h (Table 1; Figure 1B). We found variant c.527C > G (p.Ser176Trp) in exon 4 of the *TUBB8* gene by WES (Figure 3D). PROVEAN, PolyPhen-2, and SIFT predicted the missense variant as pathogenic, and according to the ACMG criteria, the variant was classified as likely pathogenic (Tables 2, 3).

In family 9, a 31-year-old woman had primary infertility for two years. In her first IVF cycle, after an early-follicular phase long-acting GnRH agonist long protocol, 10 oocytes were retrieved. Nine were at the MI stage and one was at the MII stage, but no usable embryos to transfer after fertilization (Table 1; Figure 1C). Missense variant c.763G > A (p.Val255Met) in Figure 3E, located in exon 4 of *TUBB8*, was predicted to be pathogenic by PolyPhen-2 and SIFT, and also according to the ACMG criteria, the variant was classified as a variant of likely pathogenic (Tables 2, 3).

### Mutational Spectrum and Phenotypes in *ZP1∼3*

**FIGURE 4:**
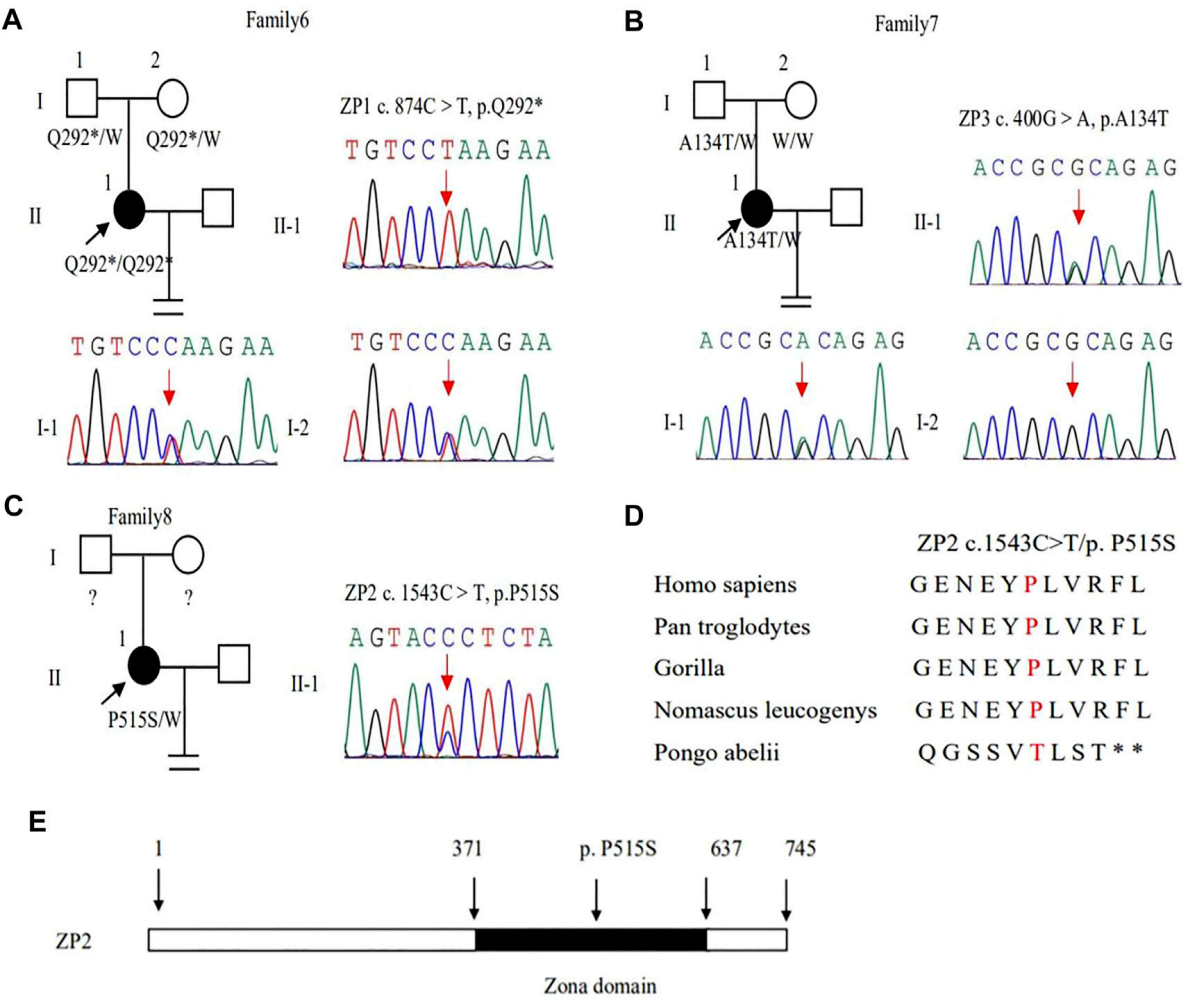
Genotypic features of *ZP1* **∼** *3*. **(A–C)** Pedigrees of the families with *ZP1* **∼** *3* variants. Sanger sequencing confirmation is shown on the right of the pedigrees. The “=” sign indicates infertility, and black circles represent affected individuals. The “W” sign means wild type, and question marks indicate the absence of a DNA sample. **(D)** Preservative mutation analysis for the novel site of *ZP2* in different species. **(E)** The position of the novel mutation is indicated in the corresponding amino acids shown on the ZP2 protein.

In family 6, a 27-year-old woman had a six-year history of primary infertility. The patient underwent two IVF/ICSI cycles. In her first cycle, using an early-follicular phase long-acting GnRH agonist long protocol, 11 follicles were monitored under the transvaginal ultrasound on the day of the human chorionic gonadotropin (hCG) trigger. However, only five oocytes were retrieved, four of which were at the GV stage (Supplementary Figure S1) and one was at the MI stage. The MI oocyte matured after IVM and formed a usable embryo, but her bloodβ-hcg test was negative two weeks after embryo transfer. In her second attempt using a midluteal short-acting GnRH agonist long protocol, seven follicles were monitored by transvaginal ultrasound on the hCG trigger day, but only two oocytes without ZP were obtained (Table 1), suggestive of EFS. A homozygous nonsense variant c.874C > T (p.Gln292^∗^) in exon 5 of *ZP1* was detected by WES. This variant was further confirmed to be inherited from her parents by Sanger sequencing (Figure 4A). PROVEAN predicted the nonsense variant to be pathogenic, and according to the ACMG criteria, the variant was classified as pathogenic (Tables 2, 3).

In family 7, a 33-year-old woman had primary infertility for three years. In her first IVF cycle using an early-follicular phase long-acting GnRH agonist long protocol, one oocyte was retrieved, which was at the MI stage. In the second attempt, two oocytes without ZP were retrieved with a midluteal short-acting GnRH agonist long protocol (Table 1; Figure 1D). We found a variant c. 400G > A (p. Ala134Thr) in exon 3 of the *ZP3* gene. This variant was validated to be paternally inherited by Sanger sequencing (Figure 4B). PROVEAN, PolyPhen-2, and SIFT predicted the missense variant as pathogenic, and according to the ACMG criteria, the variant was classified as pathogenic (Tables 2, 3).

In family 8, the patient was a 32-year-old woman with a four-year history of primary infertility. The patient underwent two IVF/ICSI cycles. In her first cycle using an early-follicular phase long-acting GnRH agonist long protocol, eight oocytes were obtained: two were at the GV stage, five were at the MI stage, and one had no ZP formed. In her second attempt with a midluteal short-acting GnRH agonist long protocol, 12 oocytes were all at the GV stage. After IVM for 24 h, seven oocytes became mature, and 3 of them developed into two-pronuclei zygotes after fertilization, nevertheless no transferrable embryos in the end (Table 1). A heterozygous missense variant c.1543C > T (p.Pro515Ser) located in exon 14 of the *ZP2* gene was detected (Figure 4C), which was not reported before. PROVEAN and PolyPhen-2 predicted the missense variant as pathogenic, and according to the ACMG criteria, the variant was classified as a variant of uncertain significance (Tables 2, 3). The conservation among primate species and the mutation location are indicated in Figures 4D,E. The results showed that mutation in *ZP2* was highly evolutionarily conserved among different primate species.

## Discussion

In our study, nine patients with oocyte maturation arrest were analyzed retrospectively, and the genetic etiology of oocyte maturation arrest was discussed. A total of nine variants in five genes, including *PATL2*, *TUBB8*, and *ZP1∼3*, were detected. All nine patients had oocyte arrest at different stages. We found three novel variants including c.1374A > G (p.Ile458Met) and 1289-1291del TCC(p.Leu430del) in the *PATL2* gene and c.1543C > T (p.Pro515Ser) in the *ZP2* gene. We also found that the patient with the variant [c.874C > T (p. Gln292*)] of *ZP1* had a lack of ZP and EFS. Altogether, these nine variants were highly conserved among different species. The variants were rare in humans with allele frequency lower than 1% in ExAC Browser.

*PATL2* gene c. 1374A > G (p. Ile458Met), which converts isoleucine to methionine, and c. 1289-1291delTCC (p. Leu430del) deletion lead to leucine deletion. The amino acid changes caused by these two variants are in the PAT1 domain (252–491amino acid), which contains 68% of the pathogenic variant of the *PATL2* gene. It is responsible for the combination with mRNA. These mutations lead to the loss of *PATL2* gene function and the decrease in the PATL2 protein expression. Because the oocyte maturation arrest type 4 is autosomal recessive inheritance, homozygous or compound heterozygous variant can lead to disease, while the patient’s mother can give birth normally. Marie Christou et al. sequenced mouse oocytes without the *patl2* gene and found that the expression of genes related to oocyte maturation was significantly downregulated (Christou-Kent et al., 2018).

The variants of *TUBB8* account for around 30% of females with oocyte maturation arrest (Feng et al., 2016a). In our study, *TUBB8* gene c. 535G > A is a missense variant that causes valine to methionine, and variant c. 527C > G leads serine to tryptophan. These two variants are located in β-tubulin subunits. Mutations at these two sites affect protein folding and stability, as well as nucleotide binding. Variant c. 686T > C transforms valine to alanine, and c. 763G > A transforms valine to methionine. These two variants are located on the surface of microtubules and may interact with microtubule-related proteins, thus interfering with the regulation and stability of microtubules (Chen et al., 2017b). The pathogenicity of these variations has been reported, consistent with the results of previous studies (Chen et al., 2019; Feng et al., 2016a; Feng et al., 2016b; Huang et al., 2017), patients with *TUBB8* mutations showed multiplicity phenotypes in oocytes and embryonic development, and the clinical characteristics of the oocytes retrieved from patients are summarized in Table1. We have five families with variations of the *TUBB8* gene, and these patients have oocytes arrested at the MI stage or have some MII oocytes that can be fertilized, such as family 9. However, there were no transferable embryos after ICSI (Feng et al., 2016a).

We found variant c. 874C > T (p. Gln292 ∗) in exon 5 in the *ZP1* gene, which results in an early termination of the codon and the termination of ZP1 protein synthesis at the 292nd amino acid encoded by exon 5. The complete ZP1 protein consists of 638 amino acids, of which there is a protein poly-nucleus composed of 279–549 amino acids, which is called the zona pellucida domain (Monné and Jovine, 2011). In humans, ZP is formed by the aggregation of ZP2, ZP3, and ZP4 proteins connected by the zona pellucida domain of ZP1 protein, and its mutation leads to premature termination resulting in ZP dysfunction (Lefièvre et al., 2004). In family 6, the patient with *ZP1* mutation was absent of ZP around the oocytes in her second cycle, and EFS was manifested in the patient. In a recent study, they found a patient with compound heterozygous mutations in *ZP1* (c.2T > A, p.M1K and c.1112+1G > T) had similar phenotype with EFS and defect in ZP (Liu et al., 2020). It suggested that the mutation of *ZP1* may play an important role in EFS, not just resulting in the absence of ZP around the oocytes (Zhang et al., 2018). The variant c. 400G > A (p. Ala 134Thr) of *ZP3* gene makes alanine to threonine, which is located in the *ZP3* domain. The domain has eight conserved cysteine residues, and it is important for protein-protein interactions (Han et al., 2010; Monné et al., 2008). Previous studies have shown that this site mutation mainly destroys the assembly of ZP, resulting in oocyte degeneration and EFS (Chen et al., 2017c; Sirard et al., 2006). However, the mutation of this site leads to the formation of oocytes without ZP in our study. The patient’s father has a heterozygous mutation, and her mother was normal, so it affected the patient’s reproductive ability through dominant-negative effects. Variant c. 1543C > T of *ZP2* changes proline into serine, which has not been reported. The point mutation is located in the zona pellucida domain of *ZP2* gene which has a role in the formation of oocytes with ZP. Homozygous and compound heterozygous mutations of *ZP2* may lead to oocyte maturation arrest in previous studies, including a thinner or absent ZP and IVF failure, following a recessive inheritance pattern. *zp2* ^−/−^ mice produced few oocytes, with the oocytes exhibiting an extremely thin ZP, and they were not fertilized (Rankin et al., 2001). Zhou et al. found that the mutated ZP2 proteins might not be secreted to the surface of the oocyte, which might lead to the formation of a thin and defective ZP (Zhou et al., 2019). A study identified two homozygous pathogenic variants (c.1695-2A > G and c.1691_1694 dup, respectively) of *ZP2* in infertile patients from two different consanguineous families: both resulted in a thin ZP and IVF failure (Dai et al., 2019). However, Yang et al. found the *ZP2* variants inherited in an autosomal dominant pattern. The heterozygous variants c.1925G > A and the heterozygous variants c.1856T > A of *ZP2* were detected in different patients with oocyte maturation arrest by WES, and they verified that heterozygous variant c.1925G > A caused altered protein modification and heterozygous variant c.1856T > A affected the intracellular transportation and secretion of ZP2 protein through *in vitro* experiments (Yang et al., 2021). We found the autosomal dominant pattern of *ZP2* in family 8 with oocyte maturation arrest as well.

However, there are several limitations of the current study. Firstly, the gDNA of parents were unavailable in some families, so we cannot determine whether the variant is *de novo* or inherited. Additionally, further functional studies should be performed to prove that these mutations affect protein function.

In conclusion, we found three novel variants in two families and analyzed the genetic causes of oocyte maturation arrest. Our study expands the spectrum of the *ZP2* and *PATL2* gene mutations. Due to the rare occurrence of these patients, future research is warranted to verify. As for the treatment of these patients, the best alternative is oocyte donation in view of this cause at present.

## Data Availability

The datasets for this article are not publicly available due to concerns regarding participant/patient anonymity. Requests to access the datasets should be directed to the corresponding author.
