# Supplementary figures and images for: Gene Spectrum and Clinical Traits of Nine Patients With Oocyte Maturation Arrest
Source: Front Genet. 2022 Jan 24;13:772143. doi: 10.3389/fgene.2022.772143 (PMC8819080; doi:10.3389/fgene.2022.772143)

**Supplementary Figure**

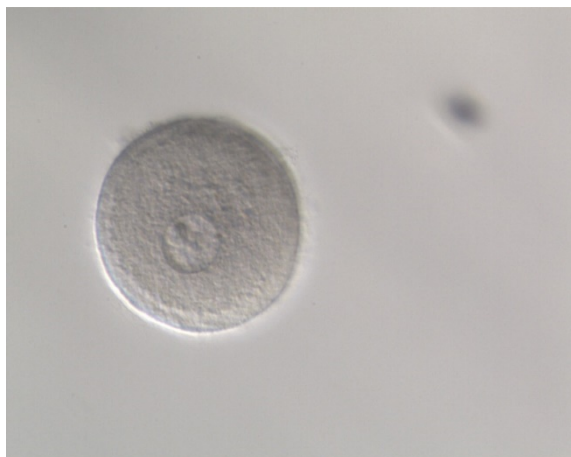

Oocyte with ZP at GV (family 6)

Supplement: Supplementary file 1 [file Image1.pdf]
